# Supplementary material for: Using excess deaths and testing statistics to improve estimates of COVID-19 mortalities
Source: medRxiv. 2021 Jan 12:2021.01.10.21249524. Preprint. [Version 1] doi: 10.1101/2021.01.10.21249524 (PMC7814852; doi:10.1101/2021.01.10.21249524)
Supplement: 1 [file NIHPP2021.01.10.21249524-supplement-1.pdf]

medRxiv preprint doi: https://doi.org/10.1101/2021.01.10.21249524; this version posted January 12, 2021. The copyright holder for this preprint (which was not certified by peer review) is the author/funder, who has granted medRxiv a license to display the preprint in perpetuity.
It is made available under a CC-BY 4.0 International license .

Supplementary Information

Examples of excess death data

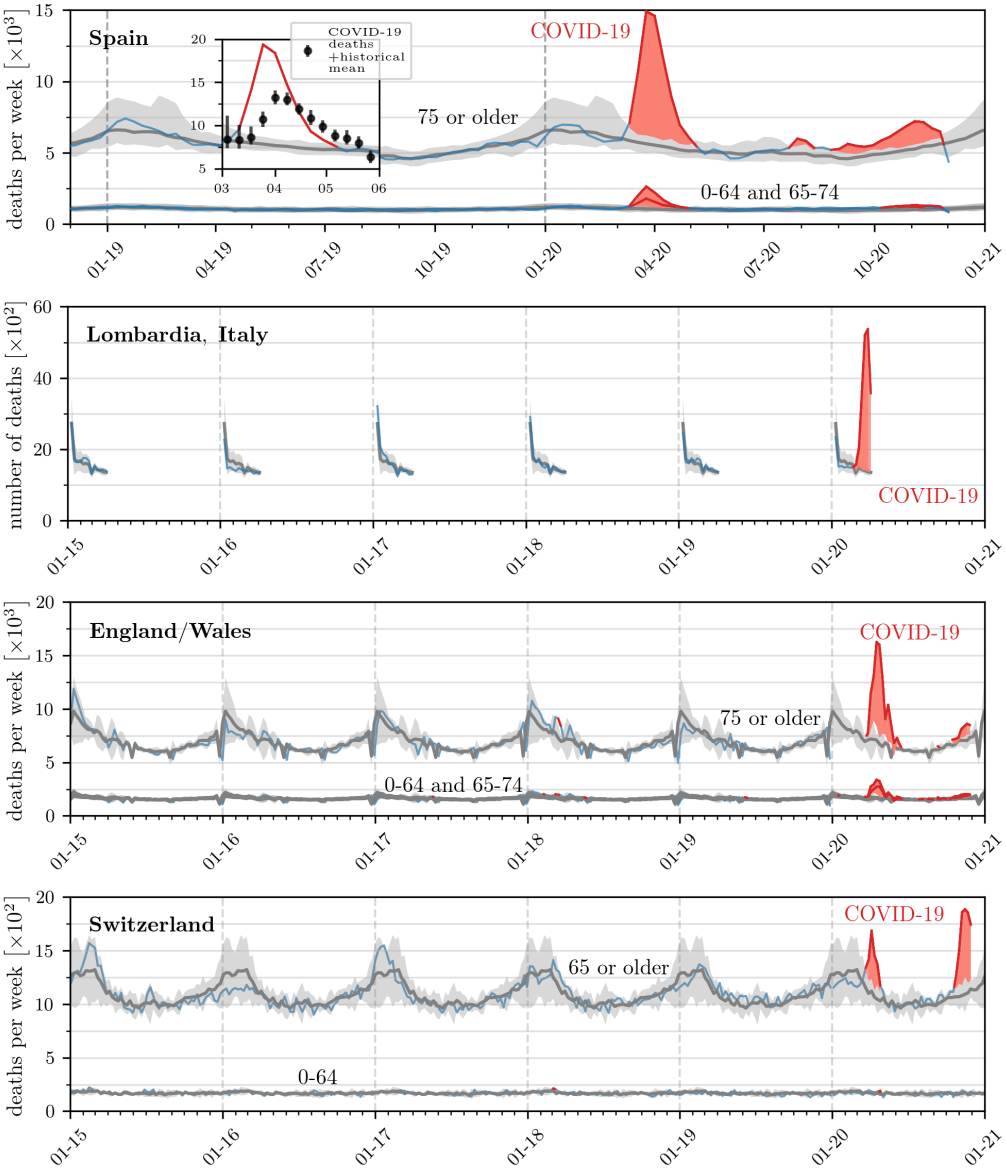

FIG. A1: **Mortality evolution in different countries**. The evolution of weekly deaths in New York City, Spain, England/Wales, and Switzerland for different age classes (where available). Grey solid lines and shaded regions represent the historical mean numbers of deaths and corresponding confidence intervals. Blue solid lines indicate weekly deaths and weekly deaths that lie outside the confidence intervals are indicated by solid red lines. For England/Wales and Switzerland, weekly means and 95% confidence intervals are based on data from 2015–2019. In the case of Spain, we show the reported COVID-19 deaths across all age classes [35] in the inset and use the 99% confidence intervals that are directly provided in the corresponding data [26]. The red shaded regions represent the mean cumulative excess deaths D_{e}. The data are derived from Refs. [21–25].

We tally weekly deaths according to Eq. (1) for each week i starting from the first week of 2020, and cumulative excess deaths as in Eq. (2) adding all weekly contributions from the first week of 2020 onwards. Note that some governmental agencies tabulate weekly deaths starting on the Sunday closest to January 1 2020 (December 29 2019, such as the United States), others instead use January 1 2020 as the first day of the week (such as Germany). A detailed list of how each country bins weekly deaths is included in Ref. [27]. The final week k up to which the cumulative count is taken depends on data availability, since some countries have larger reporting delays than others. In the majority

medRxiv preprint doi: https://doi.org/10.1101/2021.01.10.21249524; this version posted January 12, 2021. The copyright holder for this preprint (which was not certified by peer review) is the author/funder, who has granted medRxiv a license to display the preprint in perpetuity.
It is made available under a CC-BY 4.0 International license .

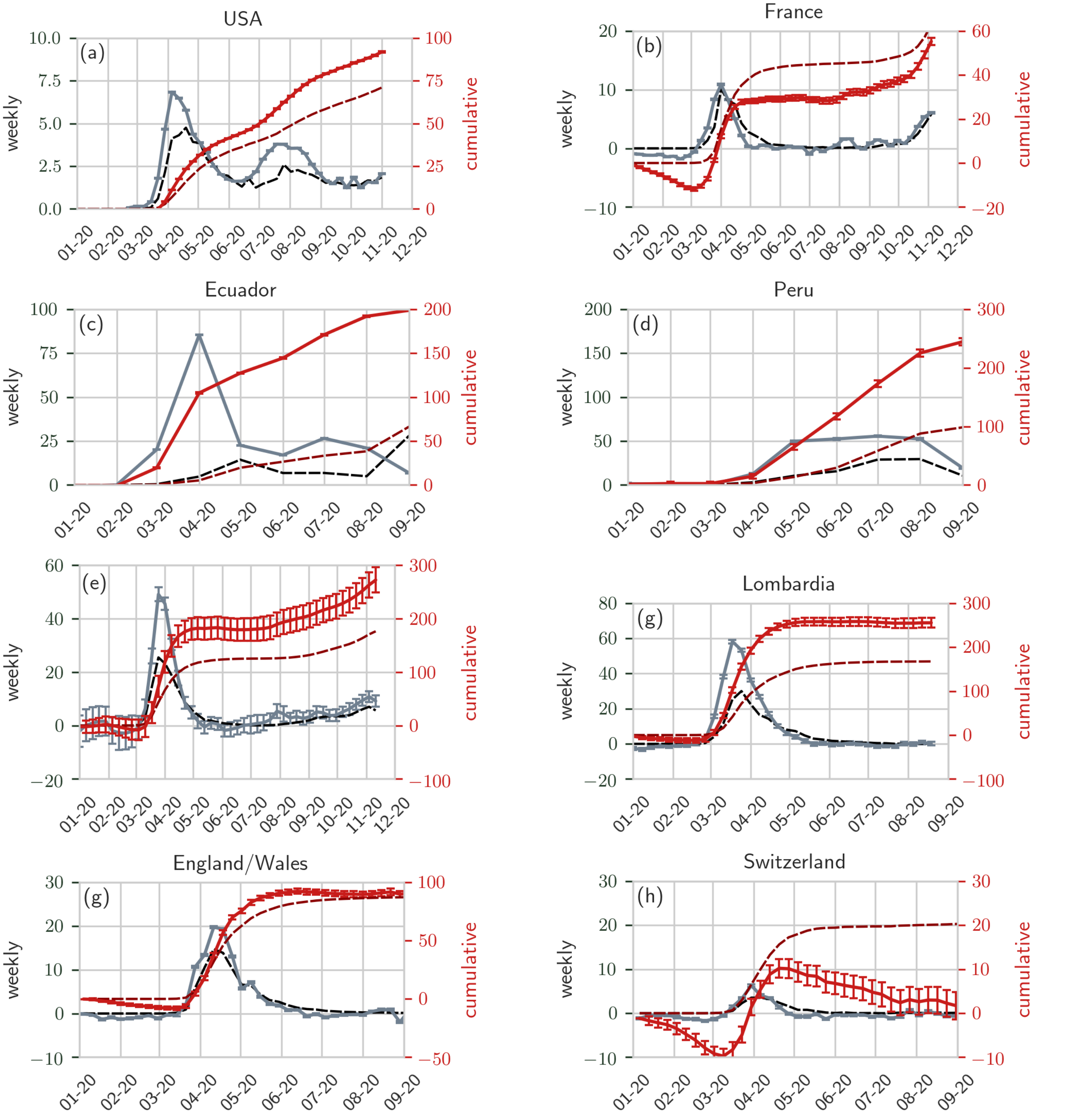

FIG. A2: **Weekly and cumulative death rates in different countries and regions**. We compare the evolution of confirmed weekly deaths $d_{\mathrm{c}}^{(0)}(i)$ (dashed black curves) and cumulative deaths $D_{\mathrm{c}}(k)$ (dashed dark red curves) with weekly excess deaths $\bar{d}_{\mathrm{e}}(i)$ (solid grey curves) and cumulative excess deaths $\bar{D}_{\mathrm{e}}(k)$ (solid red curves). The deaths are plotted in units of per 100,000 in different countries and regions. The data are derived from Ref. [27] and the error bars for the excess deaths are derived from Eqs. (1) and (2). For Spain, we used the 99% confidence intervals that are directly provided in the corresponding data [26] to approximate the 95% confidence intervals. Typically, we find $\bar{D}_{\mathrm{e}}(k) > D_{\mathrm{c}}(k)$.

of cases k is beyond the fourth week of November 2020. Quantities are calculated from data that include deaths from typically $J = 5$ previous years [27]. In Fig. A2 we plot the weekly confirmed deaths $d_{\mathrm{c}}^{(0)}(i)$, the cumulative deaths $D_{\mathrm{c}}(k) = \sum_{i=1}^{k} d_{\mathrm{c}}^{(0)}(i)$, and the mean weekly and cumulative excess deaths $\bar{d}_{\mathrm{e}}(i)$ for 2020 as available from data. We

medRxiv preprint doi: https://doi.org/10.1101/2021.01.10.21249524; this version posted January 12, 2021. The copyright holder for this preprint (which was not certified by peer review) is the author/funder, who has granted medRxiv a license to display the preprint in perpetuity.
It is made available under a CC-BY 4.0 International license .

also show $\bar{D}_{\rm e}(k)$ per 100,000 persons from the start of 2020 using Eqs. (1) and (2). The corresponding error bars in Fig. A2 indicate 95% confidence intervals defined by $\bar{d}_{\rm e}(i) \pm 1.96\,\sigma_{\rm e}(i)$ and $\bar{D}_{\rm e}(k) \pm 1.96\,\Sigma_{\rm e}(k)$ in Eqs. (1) and (2), respectively. For Spain, we used the 99% confidence intervals that are directly provided in the corresponding data [26] to approximate the 95% confidence intervals. Excess death statistics evolve differently across different countries and regions. For example, in France excess deaths were negative until the end of March 2020, quickly increasing in April 2020. In Ecuador and Peru, the number of excess deaths is more than 2.5 times larger than the corresponding number of confirmed COVID-19 deaths.

Statistical testing model

Given biases in sampling and testing errors, it is important to use a statistical testing model that takes them into account when estimating the fraction f of a population N that are infected. Testing biases arises, for example, if symptomatic individuals are more likely to seek testing. Thus, the probability $f_{\rm b}$ that an individual who chooses to be tested is positive may be different from f the probability that a *randomly* selected individual is positive, as defined in Eq. (3). If all tests are error-free, the probability that Q^+ positive results arise from the $Q \geq Q^+$ administered tests is given by

$$P_{\rm true}(Q^+|Q, f_{\rm b}) = \binom{Q}{Q^+} f_{\rm b}^{Q^+}(1-f_{\rm b})^{Q-Q^+}. \tag{A1}$$

Eq. (A1) is derived under the assumption that once individuals are tested, they are "replaced" in the population and can be tested again. The analogous distribution $P_{\rm true}(Q^+|Q, f_{\rm b})$ for testing "without replacement" can be straightforwardly derived and yields results quantitatively close to Eq. (A1) provided $Q/N \lesssim 0.3$.

Eq. (A1) also assumes flawless testing. Tests with Type I (false positives) and Type II (false negatives) may wrongly catalog uninfected individuals as infected (with rate FPR) while missing some infected individuals (with rate FNR). For serological COVID-19 tests, such as antibody tests, the estimated percentages of false positives and false negatives are typically low, with FPR $\approx 0.03 - 0.07$ and FNR ≈ 0.1 [39, 50, 51]. For RT-PCR tests, the FNRs depend strongly on the actual assay method [52, 53] and typically lie between 0.1 and 0.3 [40, 41] but might be as high as FNR ≈ 0.68 if throat swabs are used [39, 41]. FNRs can also vary significantly depending on how long after initial infection the test is administered [54]. A systematic review conducted worldwide found FNR ≈ 0.54 at initial testing [55], underlying the need for retesting. Reported percentages of false positives in RT-PCR tests are about FPR ≈ 0.05 [39]. A large meta-analysis of serological tests estimates FPR ≈ 0.02 and FNR $\approx 0.02 - 0.16$ [54]. These testing errors can lead to inaccurate estimates of disease prevalence; uncertainty in FPR, FNR will thus lead to uncertainty in the estimate of prevalence.

As illustrated through Fig. 2, errors in testing may result in the recorded number $\tilde{Q}^+$ of positive tests to be different from the Q^+ that would be obtained under perfect testing. The probability that $\tilde{Q}^+$ positive tests are returned due to testing errors can be described in terms of Q^+, FPR, and FNR and the corresponding probability distribution $P_{\rm err}(\tilde{Q}^+|Q^+, {\rm FPR}, {\rm FNR})$ is given by

$$P_{\rm err}(\tilde{Q}^+|Q^+, {\rm FPR}, {\rm FNR}) = \sum_{p_+=0}^{\tilde{Q}^+} \binom{Q^+}{p_+}(1-{\rm FNR})^{p_+}({\rm FNR})^{Q^+-p_+}\binom{Q^-}{q_+}({\rm FPR})^{q_+}(1-{\rm FPR})^{Q^- - q_+}. \tag{A2}$$

where $q_+ \equiv \tilde{Q}^+ - p_+$. By convolving $P_{\rm err}(\tilde{Q}^+|Q^+, {\rm FPR}, {\rm FNR})$ with $P_{\rm true}(Q^+|Q, f_{\rm b})$ we derive the overall likelihood distribution for the measured number $\tilde{Q}^+$ of true *and* false positives given a set of specified parameters $\theta = \{Q, f, b, {\rm FPR}, {\rm FNR}\}$ describing the population and testing

$$P(\tilde{Q}^+|Q, f, b, {\rm FPR}, {\rm FNR}) = \sum_{Q^+=0}^{Q} P_{\rm err}(\tilde{Q}^+|Q^+, {\rm FPR}, {\rm FNR}) P_{\rm true}(Q^+|Q, f_{\rm b}(f, b)). \tag{A3}$$

When $Q^+, \tilde{Q}^+$, and $Q \gg 1$, we can approximate $P_{\rm true}$, $P_{\rm err}$, and P by normal distributions and rewrite P as a function of the observed positive fraction $\tilde{f}_{\rm b} \equiv \tilde{Q}^+/Q$ (Eqs. (4) and (5)).

Using Bayes' rule, we can then formally define the likelihood of θ given a measured $\tilde{f}_{\rm b}$,

$$P(\theta|\tilde{f}_{\rm b}, \alpha) = \frac{P(\tilde{f}_{\rm b}|\theta)P_0(\theta|\alpha)}{\sum_\theta P(\tilde{f}_{\rm b}|\theta)P_0(\theta|\alpha)}, \tag{A4}$$

medRxiv preprint doi: https://doi.org/10.1101/2021.01.10.21249524; this version posted January 12, 2021. The copyright holder for this preprint (which was not certified by peer review) is the author/funder, who has granted medRxiv a license to display the preprint in perpetuity.
It is made available under a CC-BY 4.0 International license .

where $\alpha = \{\bar{\theta}, \sigma_\theta\}$ are hyperparameters defining the prior $P_0(\theta|\alpha)$, such as their means $\bar{\theta} = \{\bar{D}_{\rm e}, \overline{\rm FPR}, \overline{\rm FNR}, \bar{b}, \bar{N}\}$ and standard deviations $\sigma_\theta = \{\Sigma_{\rm e}, \sigma_{\rm I}, \sigma_{\rm II}, b, \sigma_b, \sigma_N\}$. Formally, the probability of measuring a value of a mortality measure $Z = {\rm CFR}, {\rm IFR}, M, \mathcal{M}$, or r, can be computed from

$$P(Z|\alpha) = \int P(Z|\theta)P(\theta|\alpha){\rm d}\theta, \tag{A5}$$

where $P(Z|\theta)$ defines the statistical model of the mortality measure given the components and parameters θ and the hyperparameters α defining the distribution over θ. For example, if Z is the value of the IFR, $\theta = \{D_{\rm e}, f, N\}$ and $\alpha = \{(\bar{D}_{\rm e}, \Sigma_{\rm e}), (\bar{b}, \overline{\rm FPR}, \overline{\rm FNR}, \sigma_b, \sigma_{\rm I}, \sigma_{\rm II}), (\bar{N}, \Sigma_N)\}$ are the mean and standard deviation of excess deaths, testing parameters, and the total population, respectively.

A simpler way to incorporate uncertainty in the infected fraction f is to assume a Gaussian approximation for all distributions and propagate the uncertainty in testing parameters. The squared coefficient of variation ${\rm CV}_f^2$ is then decomposed into the parameter variances according to

$$\frac{\sigma_f^2}{\hat{f}^2} \approx \frac{(1-(1-e^b)\hat{f})^2}{X^2 Q}\tilde{f}_{\rm b}(1-\tilde{f}_{\rm b}) + \frac{(1-\hat{f})^2}{X^2}\sigma_{\rm I}^2 + \frac{e^{2b}\hat{f}^2}{X^2}\sigma_{\rm II}^2 + \frac{\hat{f}^2(1-\hat{f})^2}{X^2}\sigma_b^2, \tag{A6}$$

where $X \equiv \tilde{f}_{\rm b} - {\rm FPR}$. The values of b, FPR, FNR above are mean or maximum likelihood estimates of the bias and testing errors, and σ_b^2, $\sigma_{\rm I}^2$, and $\sigma_{\rm II}^2$ are their associated uncertainties. The means and variances $\{\bar{b}, \overline{\rm FPR}, \overline{\rm FNR}, \sigma_b^2, \sigma_{\rm I}^2, \sigma_{\rm II}^2\}$ represent hyperparameters associated with testing (see SI). Our result for σ_f^2 in Eq. (A6) assumes $\{b, {\rm FPR}, {\rm FNR}\}$ are uncorrelated. Since $Q \gg 1$ is typically large, we expect the first contribution to the uncertainty, arising from stochasticity in the sampling and proportional to $\tilde{f}_{\rm b}(1-\tilde{f}_{\rm b})/Q$ to be negligible. Uncertainties in other quantities will ultimately contribute to uncertainty in the mortalities Z, as listed in Table II.

Modeling of resolved mortality

In Fig. A3, we show the evolution of $\mathcal{M}$ for Spain and Lombardia, using different effective recovery rates of unreported cases γ. We compute $\mathcal{M}$ according to Eq. (8) and use excess mortality data of Fig. A1 to determine $\bar{D}_{\rm e}$. The corresponding data for confirmed recovered and deceased individuals, $R_{\rm c}$ and $D_{\rm c}$, is taken from Ref. [26]. Current estimates of the IFR are $0.1-1.5\%$ [31–33]. To obtain a value of $\mathcal{M}$ in a similar range, we vary γ from $1-1000$ and find that $\mathcal{M} \approx 0.1-1\%$ is consistent with $\gamma = 100-1000$.

medRxiv preprint doi: https://doi.org/10.1101/2021.01.10.21249524; this version posted January 12, 2021. The copyright holder for this preprint (which was not certified by peer review) is the author/funder, who has granted medRxiv a license to display the preprint in perpetuity.
It is made available under a CC-BY 4.0 International license .

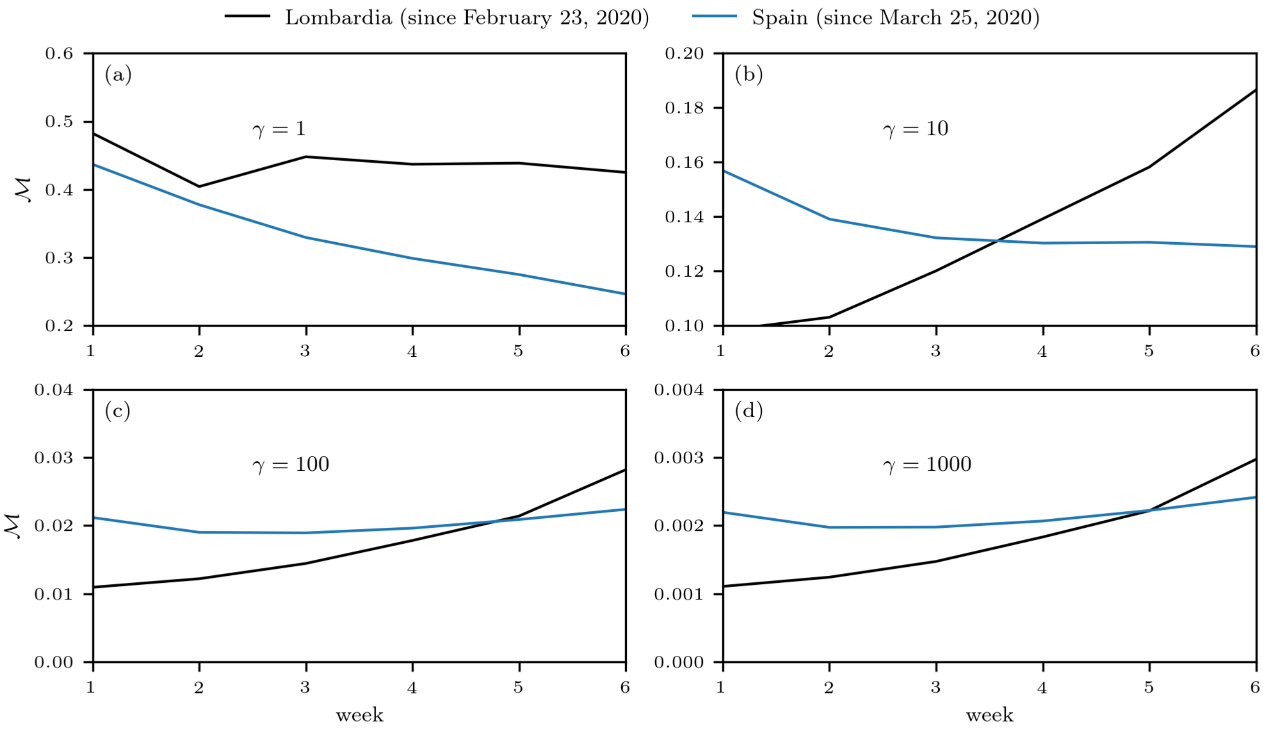

FIG. A3: **Evolution of resolved mortality.** We show the evolution of $\mathcal{M}(t)$ for different values of effective recovery rates of unreported cases γ. The data are derived from Refs. [22, 25].
